# Supplementary material for: The genome sequence of Calluna vulgaris (L.) Hull, 1808 (Ericales: Ericaceae)
Source: Wellcome Open Res. 2026 Jul 28;11:472. [Version 1] doi: 10.12688/wellcomeopenres.26654.1 (PMC13562924; doi:10.12688/wellcomeopenres.26654.1)
Supplement: Peer review history

# Reviewer report: Reviewer response for version 1

**DOI:** 10.21956/wellcomeopenres.29352.r166725

**Published:** 2026-09-18

**Authors:** Stalin Nithaniyal

**Affiliations:** 1 Botanical Survey of India, Pune, Maharashtra, India

This study presents a valuable, chromosome-level, and haplotype-resolved genome assembly for *Calluna vulgaris* (Heather), sampled from the Royal Botanic Garden Edinburgh in Scotland, UK. This ecologically important plant is widely distributed across Britain, Ireland, and northern Europe, and is characterized by diverse pollination syndromes (melittophily, myophily, and anemophily). By combining PacBio HiFi long reads (66× coverage) with Arima Hi-C scaffolding (89.67 Gb from 296.93 million reads), this study successfully resolved 97.25% of the genome (a total length of 521.83 Mb across 344 scaffolds) into 8 chromosomal pseudomolecules with a BUSCO score of 96.0%. The genomic dataset yields complete nuclear, mitochondrial (440.94 kb), and plastid (208.47 kb) sequences under the Darwin Tree of Life project. This assembly provides an initial genomic baseline to advance comparative and evolutionary analyses across the Ericaceae family.

**Advantages**

 The successful sequencing and assembly of both haplotypes (521.83 Mb and 520.42 Mb) represents a major scientific achievement, providing much deeper insights into genetic variation than traditional assemblies. Also, the combination of PacBio HiFi and Arima Hi-C technology resulted in accurate assembly continuity (Scaffold N50 of 64.34 Mb) and base accuracy (QV scores of 63.4 and 65.9). Furthermore, the complete assembly of the mitochondrion and plastid genomes provides a comprehensive dataset that will greatly facilitate future comparative genomic studies.

Are sufficient details of methods and materials provided to allow replication by others?

Yes

Is the rationale for creating the dataset(s) clearly described?

Yes

Are the datasets clearly presented in a useable and accessible format?

Yes

Are the protocols appropriate and is the work technically sound?

Yes

Reviewer Expertise:

Molecular phylogenetics

I confirm that I have read this submission and believe that I have an appropriate level of expertise to confirm that it is of an acceptable scientific standard.

# Reviewer report: Reviewer response for version 1

**DOI:** 10.21956/wellcomeopenres.29352.r166728

**Published:** 2026-09-08

**Authors:** Ruslan Kalendar

**Affiliations:** 1 University of Helsinki, Helsinki, Finland

This Data Note presents a chromosome-level, haplotype-resolved assembly of *Calluna vulgaris*, which was generated as part of the Darwin Tree of Life project. This assembly was created using PacBio HiFi (66× coverage) and Arima Hi-C data. Both haplotypes (521.83 and 520.42 Mb) have been scaffolded into eight pseudomolecules, which is consistent with the reported diploid count of 2n = 16 (Henniges et al., 2022). A total of 97.25% and 98.57% of the sequence has been assigned to chromosomes. The quality metrics are impressive: contig N50 = 2.81 Mb, scaffold N50 = 64.34 Mb, QV = 63.4/65.9, and BUSCO (eudicots_odb10) = 96.0%, with only 5.3% being duplicates. The combined k-mer completeness is 99.21%, and the EBP score is 6.C.Q63. Organellar genomes (mitochondrion: 440.94 kb; plastid: 208.47 kb) are also provided. The methods follow the well-documented ToL pipelines and the software versions are tabulated. All data have been deposited in the ENA under the accession numbers PRJEB74151 and GCA_964145725.1/GCA_964145215.1. This is a valuable resource for a keystone heathland species, as well as for comparative genomics in the Ericaceae family (e.g. Vaccinium and Rhododendron).

 The work is technically sound, and the data are presented clearly. I have only minor comments that the authors may wish to address in a revised version:

 1. Inconsistency regarding haplotype 2. The 'Assembly statistics' section states that 'haplotype 2 was assembled to scaffold level', whereas the abstract, Table 2 (assembly level: chromosome) and Table 3 indicate that haplotype 2 has also been scaffolded into eight chromosomal pseudomolecules (98.57%). Please harmonise the text.

 2. Genome size estimates. Flow cytometry gives 1C = 0.63 pg (620 Mb), GenomeScope2 gives 544 Mb and the assembled haplotypes give 521 Mb. The 15% discrepancy between the cytometric and assembly-based estimates is not unusual for a genome with 44% repeat content. However, a sentence acknowledging this discrepancy (e.g. due to collapsed repeats/rDNA arrays) would be helpful.

 3. Ploidy of the sequenced individual. Since triploid and tetraploid individuals have been reported (Behrend et al., 2015), it would be useful to explicitly state that the k-mer spectrum (with two clear peaks, p = 2) and the 1C-value confirm that the ddCalVulg4 specimen is diploid.

 4. Table 4. The k-mer completeness benchmark (≥95%) is presented alongside the per-haplotype values (74%), as would be expected for a phased diploid assembly. However, a note clarifying that the benchmark applies to the combined haplotypes (99.21%) would help to avoid any potential confusion.

 5. Figure 3. A conspicuous grey vertical/horizontal band is present at 150 Mb (the boundary between chromosomes 2 and 3) in the Hi-C contact map. Please clarify whether this is a rendering artefact of PretextSnapshot or whether it reflects a region of low Hi-C mapping. Consider regenerating the figure.

 6. Figure 6 (blob plot). Eight scaffolds (310 kb) are assigned to Ascomycota, while 326 scaffolds (19.1 Mb) have no hit. Please state whether the Ascomycota-assigned scaffolds were removed from the released assembly or retained and explain why. The difference in scaffold count between Figure 5 (346) and Table 2 (344) presumably reflects the two organellar contigs. A brief note would be helpful here.

Are sufficient details of methods and materials provided to allow replication by others?

Yes

Is the rationale for creating the dataset(s) clearly described?

Yes

Are the datasets clearly presented in a useable and accessible format?

Yes

Are the protocols appropriate and is the work technically sound?

Yes

Reviewer Expertise:

bioinformatics and computational genomics

I confirm that I have read this submission and believe that I have an appropriate level of expertise to confirm that it is of an acceptable scientific standard.

# Reviewer report: Reviewer response for version 1

**DOI:** 10.21956/wellcomeopenres.29352.r162953

**Published:** 2026-09-04

**Authors:** Stephen P. Moose

**Affiliations:** 1 University of Illinois Urbana-Champaign, IL, USA

This article describes a nearly complete genome assembly for *Calluna vulgaris* (heather), a plant found widely throughout Britain, Ireland, and northern Europe.  Flow cytometry estimated the haploid genome size at 620Mb, and 544.2Mb were recovered in the assembly.  The genome was assembled into two haplotypes of 2.81 and 2.56Mb, each with 8 chromosomes in 20-50 scaffolds.  The genome sequence is high-quality, exceeding benchmark metrics for k-mer completeness, BUSCO analysis of gene content, and chromosome coverage (97.25%).  The individual sequenced is preserved at the Royal Botanical Garden Edinburgh and the genome assembly is deposited at the European Nucleotide Archive.

Are sufficient details of methods and materials provided to allow replication by others?

Yes

Is the rationale for creating the dataset(s) clearly described?

Yes

Are the datasets clearly presented in a useable and accessible format?

Yes

Are the protocols appropriate and is the work technically sound?

Yes

Reviewer Expertise:

Plant genomics, I have directed whole-genome sequence and assembly projects.  Thus, I am qualified to review this work.

I confirm that I have read this submission and believe that I have an appropriate level of expertise to confirm that it is of an acceptable scientific standard.
